# A multidisciplinary dermatology-gastroenterology-rheumatology (DER.RE.GA) unit for the care of patients with immune-mediated inflammatory diseases: analysis of the first 5 years from the dermatologist’s perspective

**DOI:** 10.3389/fmed.2023.1290018

**Published:** 2023-11-30

**Authors:** Valeria Brazzelli, Francesca Bobbio Pallavicini, Paolo Maggi, Łukasz Chętko, Eugenio Isoletta, Nicolò Di Giuli, Alice Bonelli, Valentina Fornaroli

**Affiliations:** ^1^Institute of Dermatology, Fondazione IRCCS Policlinico San Matteo, Pavia, Italy; ^2^Department of Clinical, Surgical, Diagnostic and Pediatric Sciences, Institute of Dermatology, Università degli Studi di Pavia, Pavia, Italy; ^3^Rheumatology Department, Fondazione IRCCS Policlinico S. Matteo, Pavia, Italy; ^4^Student Scientific Research Club of Experimental, Clinical and Procedural Dermatology, Medical University of Lodz, Łódź, Poland

**Keywords:** psoriatic arthritis, seronegative arthritis, inflammatory bowel disease, psoriasis, multidisciplinary unit

## Abstract

Immune-mediated inflammatory diseases (IMIDs) constitute a heterogenous group of chronic and highly disabling conditions. The clinical challenges they often pose led to formation of numerous dermo-rheumatological interdisciplinary units around the world, which are reported to benefit their patients in various ways. The present paper describes our experience with a multidisciplinary dermatology-rheumatology-gastroenterology unit DERREGA at the IRCCS Foundation Policlinico San Matteo of Pavia over a period of 5 years of its activity (2017–2022). A digital database was created, containing the medical records of 146 patients referred to the dermatology unit only by rheumatologists or gastroenterologists belonging to the multidisciplinary unit DERREGA. Then, aspects such as demographics, initial basis of referral and final diagnosis among the patients were analyzed retrospectively. Patients were classified as either gastroenterological or rheumatological, and then categorized according to the specific basis of referral. Most of the gastroenterological patients (97%) were affected by inflammatory bowel diseases (IBDs). Rheumatological patients were divided in three subgroups, including patients referred with vasculitis, arthropathies (undifferentiated arthritis, psoriatic arthritis and other arthritis) and other rheumatological diseases. Then, final diagnoses were evaluated in each group. Almost a third of IBD patients received a diagnosis of paradoxical psoriasis. Dermatological examination allowed diagnosis of minimal psoriasis based on Caspar criteria in over 70% of the patients admitted with undifferentiated arthritis. A multidisciplinary approach is suggested to provide more effective management of IMIDs and, specifically, from a dermatological perspective, allows for the diagnosis of minimal manifestations of psoriasis in patients with a provisional diagnosis of undifferentiated arthritis.

## 1 Introduction

Over the last three decades, the progress and development of medicine in terms of diagnostic, laboratory and therapeutic knowledge, along with better understanding of genetics and immunogenetics, reinforced the significance of collaboration among different specialties. According to experiences reported around the world, the multidisciplinary approach allows to improve the diagnosis and treatment of various immune-mediated inflammatory diseases (IMIDs) and therefore enables to improve patients’ compliance as well as health related quality of life (HRQOL).

The synergy between dermatology and rheumatology has long been recognized and led to the foundation of numerous interdisciplinary clinics. These units try to co-manage different immune-mediated diseases, such as, primarily, psoriasis and psoriatic arthritis (PsA), by avoiding unnecessary tests; provide more effective treatment, by using systemic and biologic therapies and by avoiding treatment associated risks; reduce costs and achieve better disease control, which contributes to better HRQOL (1, 2).

An interdisciplinary dermatologic-rheumatologic-gastroenterological approach, however, is by far less common and the data available in literature are meager.

Nevertheless, IMIDs, including autoimmune diseases, are extremely frequent and affect up to 10% of western population. In fact, they encompass inflammatory bowel diseases (IBD), such as Crohn’s disease and ulcerative colitis; psoriasis and PsA; hidradenitis suppurativa; connective tissue diseases and systemic lupus erythematous (3). Besides, there exists evidence indicating that patients affected by one IMID face greater probability of developing multiple IMIDs (4). The constantly changing immune imbalance associated with IMIDs increases the difficulty of treatment (5). Lack of proper communication and cooperation between healthcare professionals (HCPs) often leads to discrepancies concerning therapeutic goals and outcome measures as well as to less regular follow-up. Given the factors mentioned above, providing high-quality management of patients with IMIDs remains a challenge (6).

The aim of the following, retrospective, cohort study is to describe our experience with the interdisciplinary dermatology-rheumatology-gastroenterology unit “DERREGA” at the IRCCS Foundation Policlinico San Matteo of Pavia over a period of 5 years, between December 2017 and December 2022. In light of the literature published to date, we supposed that collaboration between the clinicians involved would provide the patients with more benefits in comparison with a traditional, siloed approach.

## 2 Materials and methods

The need for improvement in diagnosis and treatment of rheumatological, gastroenterological and dermatological IMIDs led to the formation of the interdisciplinary dermatology-rheumatology-gastroenterology unit called “DERREGA” at the IRCCS Foundation Policlinico San Matteo of Pavia in 2017. The objectives were to facilitate the diagnosis in rare and complex cases by involving diverse clinicians in the diagnostic process; to improve the management of the disease and dermatological symptoms in patients difficult to treat; to provide shared therapeutic choice in order to reduce the number of drugs administered along with the number and the entity of subsequent adverse events, and thereby improve patients’ compliance and HRQOL.

The unit DERREGA is based on shared agendas, by means of which different specialists can schedule appointments either once a week or according to the needs of both patients and HCPs. Besides this clinical activity, a specialist can also request consultations or meetings, held in order to discuss the most difficult and problematic clinical cases.

The selection criterion for admitting a patient to DERREGA unit was the presence of at least one rheumatological, dermatological or gastroenterological IMID, which demanded evaluation by at least one other specialist due to its complex nature.

In the course of the following, retrospective, cohort study, we evaluated a total number of 146 dermatological patients who met the eligibility criteria and were referred to DERREGA unit in the period of its activity between December 2017 and December 2022. The medical records of the patients were converted into a digital database including the following information:

- sex
- age and date of birth
- referring specialist
- disease in anamnesis
- basis of referral
- potential drug use
- comorbidities
- final diagnosis

On the basis of the disease in anamnesis and the referring specialist or department, the patients were divided into two main groups, the ones referred by gastroenterologists and the ones referred by rheumatologists.

The patients referred by gastroenterologists were further divided into the ones affected with chronic IBDs and the ones suffering from other gastrointestinal pathologies, likely to present cutaneous manifestations. The former group was subdivided into patients affected with Crohn’s disease and the ones with diagnosis of ulcerative colitis.

The patients referred by a rheumatologist, however, were subdivided in groups based on their underlying disease: vasculitis (group A), arthropathies (group B) and other rheumatological diseases (group C). Then, group B was divided into patients afflicted with undifferentiated arthritis, PsA and other arthropathies.

**Figure 1:**
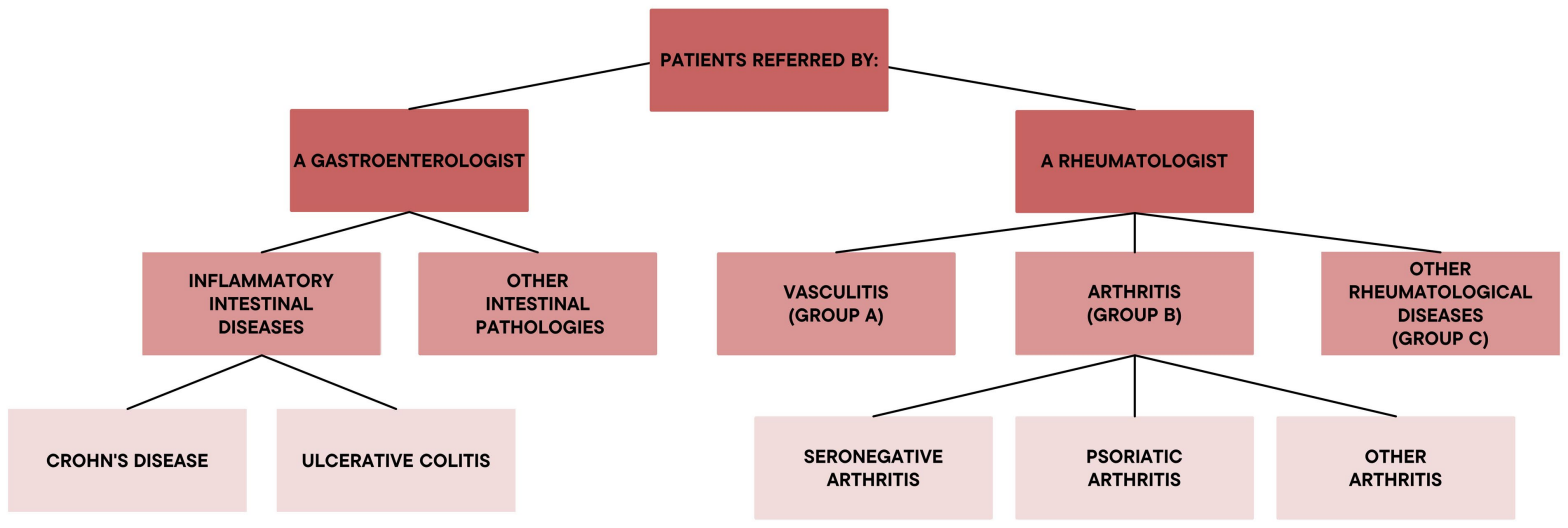
Functional groups of the dermatological referrals.

Finally, the gathered data, including the final diagnoses, were evaluated in each group (Figure 1).

## 3 Results

In the period between December 2017 and December 2022, the multidisciplinary team of the dermatological department in the combined clinic DERREGA evaluated a total number of 146 patients with an average age of 53.9 years. Among these 146 patients, 79 (54.1%) were female, with an average age of 54.6 years and 67 (45.9%) were male, with an average age of 53 years. The majority of the patients (111/146), constituting 76%, were referred to the unit by rheumatologists, whereas 35 out of 146 patients, corresponding to 24%, were referred by gastroenterologists.

### 3.1 Gastoenterological patients

**Figure 2:**
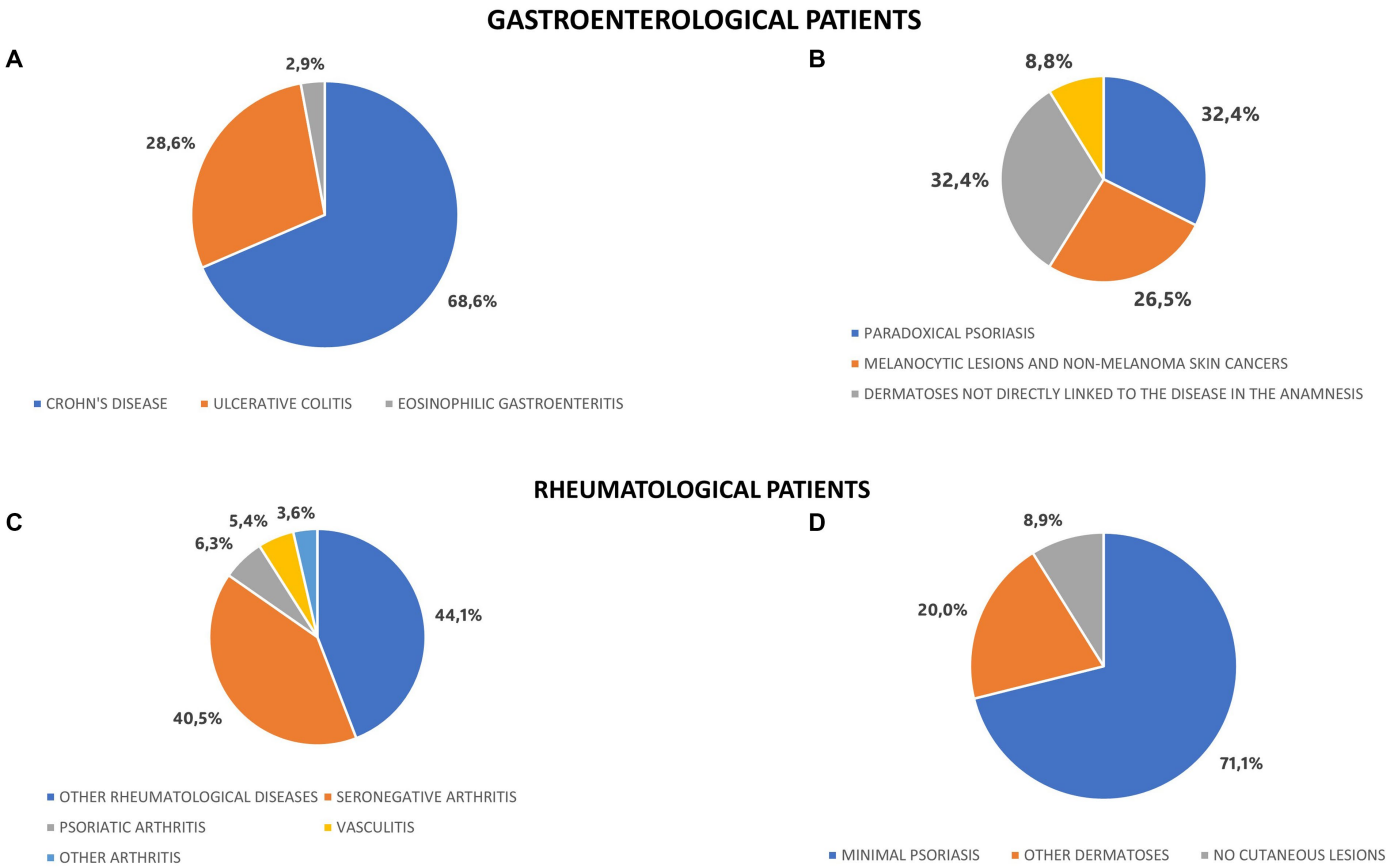
Underlying diseases and final diagnoses in gastroenterological and rheumatological patients. **(A)** Disease in anamnesis among gastroenterological patitents. **(B)** Final dermatological diagnoses in patitents affected by IBDs. **(C)** Disease in anamnesis among rheumatological patients. **(D)** Final dermatological diagnoses in patients affected by undifferentiated arthritis.

Among the patients referred by gastroenterologists, 34 out of 35 (97%) were affected by inflammatory bowel diseases (IBDs). Among this group, 24 out of 35, corresponding to 68.6%, were affected by Crohn’s disease and 10 patients out of 35, corresponding to 28.6%, were affected by ulcerative colitis. Only 1 out of 35 patients (3%) was affected by eosinophilic gastroenteritis and referred to the DERREGA unit in order to exclude potential cutaneous involvement (Figure 2A).

The final diagnoses of the 34 patients affected by inflammatory bowel diseases involved dermatoses linked to adverse events of the ongoing therapy, which occurred in 12 subjects, corresponding to 35.3%. Essentially, paradoxical psoriasis was the most common diagnosis in this group and concerned 11 of the patients, constituting around one third of all the evaluated subjects with IBDs.

Another 9 patients, equal to 26.5%, were diagnosed with melanocytic lesions and non-melanoma skin cancers. The remaining 11 patients, corresponding to 32.4%, presented dermatoses which were not directly linked to the disease in anamnesis (Figure 2B).

### 3.2 Rheumatological patients

A total of 111 patients (76%) were referred to DERREGA unit by rheumatologists. Group A included 6 out of 111 patients, corresponding to 5.4%, who had already been diagnosed with vasculitis and were referred to the unit in order to achieve better control and management of the dermatologic symptoms. Group B involved 56 out of 111 patients, corresponding to 50.5%, who were affected by arthritis. Group C comprised 49 out of 111 patients, equal to 44.1%, who were affected by other rheumatological diseases, including connective tissue diseases, lupus erythematosus and chronic IMIDs.

The patients affected by arthritis (Group B) were further divided into the following groups: 45 patients affected by undifferentiated arthritis; 7 patients affected by PsA, who were referred to the unit in order to achieve a better control and treatment of the cutaneous psoriasis; 1 patient affected by ankylosing spondylitis and 3 patients affected by rheumatoid arthritis, who were referred to the unit due to a dermatosis that was not directly interconnected with the rheumatological disease in anamnesis (Figure 2C).

Forty-five patients affected by undifferentiated arthritis were referred to the DERREGA unit for dermatological evaluation in search of minimal signs of psoriasis. Essentially, 32 out of 45 patients, corresponding to 71.1%, were finally diagnosed with minimal psoriasis and consequently with PsA. 9 out of 45 patients (20%) were diagnosed with dermatoses which were not interconnected with undifferentiated arthritis present in anamnesis and 4 out of 45 patients (8.9%) did not present any dermatological signs or symptoms (Figure 2D).

Forty-nine patients affected by other rheumatological diseases, ascribed to group C, were referred to the unit for three main reasons: simultaneous presence of dermatological and rheumatological symptoms in 8 of the subjects, corresponding to 16.3%; need of better control and treatment of dermatological symptoms of the rheumatologic diseases in anamnesis in 28 of the patients, constituting 57.1%; need of evaluation of present dermatological symptomatology not directly linked to the rheumatological disease in the anamnesis in 13 of the patients, equal to 26.5%.

## 4 Discussion

According to the available literature, involvement of multidisciplinary teams in the treatment of IMIDs displays manifold benefits for the patients. Easier communication between specialists enables more efficient and accurate diagnostic process with less delayed decision making. Furthermore, shared therapeutic choice is more likely to provide the patient with optimized and more effective treatment, thanks to better access to novel therapies and improved safety when using immunosuppressants for various indications. This contributes to overall improvement in patient satisfaction and QoL. Besides, the patient is given reassurance that issues most important to them are being considered and addressed. Consequently, patients are likely to become more involved and adherent in their own care (7).

Due to its numerous advantages, multidisciplinary care is considered valuable and widely recommended in the management of IMIDs. The 2019 update of the EULAR recommendations for the management of PsA emphasizes the necessity of collaboration between a rheumatologist and a dermatologist in the presence of clinically significant skin involvement (8). The 2021 update to GRAPPA recommendations also advocates a multidisciplinary approach to PsA management (9). Nonetheless, validated and standardized strategies aimed at integrated and holistic approach to IMIDs are non-existent or only beginning to emerge in some settings, including spondyloarthropathy (SpA), IBDs and psoriatic disease. An attempt to determine a core set of statements, concerning the management of SpA-related IMID, was made by Rizzello et al., who published the consensus regarding the principles of cooperation between a rheumatologist, dermatologist, gastroenterologist and ophthalmologist in 2018 (2). Apart from that, in 2022 Cusano et al. proposed the potential organization of a combined Dermatology-Rheumatology unit in Italy (10).

As far as psoriasis and PsA are concerned, several retrospective studies already report superiority of combined dermo-rheumatological clinics over siloed approach in terms of effectiveness in both diagnosis and treatment (11–17). Observations from the management of psoriasis and PsA in the multidisciplinary dermatology-rheumatology clinic described by Velez et al. in 2012 indicate that, during a 6-year period, a significant number of patients received a revised diagnosis which differed from the one initially obtained at other centers (11). Furthermore, several studies point out easier access to systemic medication and biologics in combined clinics along with more timely treatment initiation (11–15).

To our knowledge, however, only one study conducted by Hjuler et al. is currently aimed at determining the effectiveness of a combined dermato-rheumato-gastroenterological clinic so far. Nonetheless, no results have been published to date (3).

One of the most notable advantages of the DERREGA unit with regard to the population of gastroenterological patients was more effective diagnostic process, which resulted in recognition of paradoxical psoriasis in 11 subjects, constituting 30% of their number. There exist several publications considering the possible correlation between the presence of an IBD in anamnesis, treatment with an anti-TNF biologic and the occurrence of psoriasiform cutaneous lesions. The results from our study seem to be in line with some of them.

In 2011, Cullen et al. published a cohort metaanalysis, evaluating a group of 120 patients affected by an IBD and treated with an anti-TNF biologic, who had been described in the literature. It revealed that 30 patients, constituting 25% of all the evaluated subjects, presented the clinical image of paradoxical psoriasis (18).

In 2020, Bucalo et al. published a study in which paradoxical psoriasis turned out to affect 16 out of 53 evaluated patients (30.2%) suffering of an IBD and treated with an anti-TNF biologic drug. Additionally, the authors suggested the possible presence of genetic predisposition to the development of paradoxical psoriasis. In fact, the study revealed more frequent occurrence of the TNF-α rs1799964 allele, and less frequent occurrence of the HLAcW6 allele among the patients presenting paradoxical psoriasis (19).

Secondly, a prominent number of patients was admitted to the DERREGA unit in order to monitor the melanocytic lesions both prior to biological therapy and after the beginning of the treatment. In this case, dermatological evaluation was aimed at early detection of potential melanoma. Nevertheless, throughout the 5 years of the activity of the unit, no melanomas were diagnosed. As far as non-melanoma skin cancer diagnoses are concerned, only one of the patients was diagnosed with basal cell carcinoma.

These results appear to be in line with those published by Esse et al. in 2020 (20). The authors compared a cohort of 34,029 patients with IBDs, treated with biologic agents, with a cohort of 135,370 patients affected with IBDs, treated with conventional, systemic therapies. In the end, no statistically significant differences in terms of the occurrence of melanoma between the evaluated groups were noted, which indicated that there is no evidence proving the correlation between biological therapy and increased risk of occurrence of melanoma in the patients.

Finally, as IMIDs share common inflammatory pathways involved in their pathogenesis, it appears that some of the biologics are likely to act simultaneously on an IBD and psoriasis coexisting in one patient (21). This aspect can reinforce the significance of shared therapeutic choice aimed at providing safer and more beneficial treatment of IMIDs (7).

The activity of the unit DERREGA contributed to improving diagnosis in the population of rheumatological patients too. Essentially, dermatological evaluation enabled the detection of minimal psoriasis in 32 out of 45 patients admitted with undifferentiated arthritis, corresponding to 71.1%. The evidence of minimal signs of psoriasis in this group thereby allowed early diagnosis of PsA on the basis of CASPAR criteria. Consequently, the treatment targeting both cutaneous and articular symptoms could be initiated more timely. This resulted in better prognosis and improved HRQoL.

Currently, no PsA-specific diagnostic test is available (12). Nevertheless, cutaneous lesions precede the development of PsA in around 80% of the cases (22). Furthermore, localization of minimal psoriasis in specific areas such as scalp, nails, perianal or intergluteal region is known to increase the risk of developing PsA (23). Therefore, cursory evaluation of the patient’s skin on rheumatological examination can delay the diagnosis of PsA in its early stages (12). Besides, uncommon clinical image of already present cutaneous psoriasis can make the diagnosis based on CASPAR criteria particularly difficult for a rheumatologist (24). On the other hand, however, there is no correlation between the severity of cutaneous manifestations and the presence of arthritis and around 20% of the patients develop PsA prior to the cutaneous lesions (11). Consequently, suspicion of PsA may not always be obvious also for a dermatologist (12). In fact, it is estimated that between 10 and 29% of the patients with psoriasis assessed by a dermatologist can be underdiagnosed. For these reasons, collaboration between the two specialists and careful monitoring of the skin condition plays a significant role in early detection of PsA, which is likely to prevent further joint damage and long-terms disability.

Our results appear to share some similarities with the experiences from several dermo-rheumatological units already described in the literature. According to Velez et al., during 6 years of the activity of a combined dermo-rheumatological unit in Boston, MA, US, 53% of the patients admitted with joint pain were eventually diagnosed with PsA. The authors claim that these diagnoses may not have been easily distinguished in a traditional clinical setting (11). Increase in the number of patients diagnosed with PsA and thereby receiving appropriate treatment was also observed in a combined dermo-rheumatological unit in Italy, described by Foti et al. (13). Luelmo et al. report that in a combined unit of psoriasis of PsA in Spain, almost half of the cases of concomitant PsA were diagnosed *de novo*, due to the suspected diagnosis of a dermatologist. Besides, the unit enabled successful differential diagnosis of PsA from other conditions. Interdisciplinary collaboration led to changes in the initial clinical conclusion in almost one third of all the evaluated patients and to modifications of treatment in over half of them (12).

Secondly, DERREGA contributed to more effective and accurate diagnosis in 9 out of 45 patients (20%) with undifferentiated arthritis, as it enabled recognition of other dermatoses, not linked to the disease in anamnesis. Thanks to it, this group could obtain the necessary treatment more quickly. Similarly, in the unit described by Luelmo et al., over 10% of the patients admitted were eventually diagnosed with other skin diseases (12). Although the remaining 4 patients (8.9%) with undifferentiated arthritis did not present any cutaneous lesions at all, this information, confirmed by a dermatologist, may still turn out to be of use for a rheumatologist in some cases of diagnostic doubts. Specialists from 20 combined dermo-rheumatological clinics in the US, including Stanford and Harvard, admit unequivocally that multidisciplinary approach enabled more prompt and accurate diagnoses, resulting in timely interventions and improved outcomes (25).

Furthermore, the unit DERREGA benefited 7 out of 111 patients, equal to 6%, who had already been diagnosed with PsA prior to the admission. In this case, interdisciplinary collaboration between a dermatologist and a rheumatologist provided more effective control of cutaneous and articular symptoms. Besides, involvement of both specialists in the therapeutic choice enabled to maximize the effects of the treatment and decrease the occurrence of adverse events, and thereby improve the HRQOL in this group.

Actually, in the Spanish combined clinic, described by Urruticoechea-Arana et al., cutaneous symptoms difficult to manage constituted the most common reason for the admission. Multidisciplinary evaluation resulted in changes in systemic treatment in 42% of the patients and more frequent administration of topical treatment. The final level of satisfaction achieved among the patients is reported to have been high (14). Similar experiences are presented by Pérez-Barrio et al., who conducted a metanalysis of 188 patients with psoriasis and moderate-to-severe PsA. It revealed that 44% of the necessary changes in treatment resulted from insufficient control of the cutaneous symptoms. In turn, the modifications instituted by the interdisciplinary team produced an improvement and led to eventual discharge in the majority of the patients (26). In 2018, Luchetti et al. published the algorithm adopted in a combined dermo-rheumatological clinic, which enabled effective recognition, classification and therapeutic choice in PsA. Evaluation of a cohort of 116 patients with PsA showed significant increase in the measures of both HRQOL scale and dermatological life quality index (DLQI) after 48 months of treatment (15). High levels of overall patient satisfaction from combined dermato-rheumatological care were also proved by the results of a survey conveyed by Foulkes et al. (16).

As far as other rheumatological diseases are concerned, in the light of the literature published to date, multidisciplinary approach appears to have benefited the patients in manifold ways. In fact, 8 of the patients (16.3%) required collaboration between specialists in order to achieve the correct final diagnosis due to the coexistence of dermatological and rheumatological symptoms. The complexity of clinical manifestations of various rheumatological diseases can be reflected in the variety of conditions seen in the combined dermo-rheumatological unit described by Argobi et al. The authors point out frequent diagnostic doubts concerning cutaneous symptoms and the tendency for siloed approach among both dermatologists and rheumatologists. Surprisingly, the study showed that despite the presence of cutaneous manifestations of the disease in history of all of the patients, only 34% of them had been evaluated by a dermatologist prior to the admission. Besides, an even smaller number had had these concerns addressed by a rheumatologist. These results can prove that multidisciplinary clinical setting provides a chance for better management of the already diagnosed disease in the patients with insufficient control of cutaneous manifestations. Lastly, it is noteworthy that around 30% of the patients admitted to the mentioned unit received secondary diagnoses which made a significant difference in the management (17). This tendency can support the benefits from interdisciplinary assessment in 13 of the patients (26.5%) in the group C, who similarly demanded evaluation of dermatoses indirectly linked to the primary diagnosis.

### 4.1 Limitations

Despite numerous merits displayed by multidisciplinary units, several challenges still remain. As such institutions do not usually function on a daily basis, due to the limited availability of the specialists, it may be difficult to coordinate the available number of appointment slots with patients’ schedules. This may contribute to less frequent follow-up and consequently limit the potential benefits. Furthermore, the complexity of their formal administration and technical organization as well as different duration times of dermatological and rheumatological visits may constitute obstacles preventing such units from effective functioning. These factors, along with time consumption and cost-ineffectiveness may make it difficult to convince the institution of the real “added value” of multidisciplinary units or event prevent their formation (26, 27).

### 4.2 Conclusion

The combined unit DERREGA has benefited both rheumatological and gastroenterological patients in various ways throughout the 5 years of its activity. Primarily, it enabled their more effective dermatological evaluation, which facilitated the diagnostic process significantly. Besides, multidisciplinary approach contributed to more effective control and treatment of cutaneous symptoms both directly and indirectly associated with the underlying disease. Involvement of specialists from various areas of medicine contributed to a decrease in the number of administered drugs and occurrence of subsequent adverse events. This resulted in better compliance among the patients and improvement in terms of their HRQoL. Given the benefits mentioned above, the results from our study can reinforce the significance of providing multidisciplinary care in patients with IMIDs and support the effort aimed at encouraging collaboration between clinicians.

## Data availability statement

The raw data supporting the conclusions of this article will be made available by the authors, without undue reservation.

## Ethics statement

Ethical approval was not required for the studies involving humans because the study protocol did not deviate from standard clinical practice. The studies were conducted in accordance with the local legislation and institutional requirements. The participants provided their written informed consent to participate in this study.

## Author contributions

VB: Conceptualization, Data curation, Investigation, Writing – original draft, Writing – review & editing, Supervision. FB: Conceptualization, Investigation, Data curation, Writing – original draft. PM: Data curation, Investigation, Writing – original draft. ŁC: Writing – original draft, Writing – review & editing. EI: Conceptualization, Data curation, Writing – review & editing. NG: Writing – review & editing. AB: Data curation, Investigation, Writing – original draft. VF: Writing – review & editing.
